# Retraction Note: Alteration of astrocytes and Wnt/β-catenin signaling in the frontal cortex of autistic subjects

**DOI:** 10.1186/s12974-016-0568-9

**Published:** 2016-05-13

**Authors:** Fujiang Cao, Ailan Yin, Guang Wen, Ashfaq M. Sheikh, Zujaja Tauqeer, Mazhar Malik, Amenah Nagori, Michael Schirripa, Frank Schirripa, George Merz, Shiqing Feng, W. Ted Brown, Xiaohong Li

**Affiliations:** Department of Neurochemistry, NY State Institute for Basic Research in Developmental Disabilities, 1050 Forest Hill Road, Staten Island, NY USA; Department of Developmental Neurobiology, NY State Institute for Basic Research in Developmental Disabilities, New York, NY 10314 USA; Digital Microscopy, NY State Institute for Basic Research in Developmental Disabilities, New York, NY 10314 USA; Department of Orthopaedics, General Hospital of Tianjin Medical Universtiy, Tianjin, China

The corresponding author Xiaohong Li is retracting this article [1]. Following a review of the original data, the results presented in Figures 5A and 5B which underpin the conclusions of this study were found to be unreliable because of possible technical artifacts in lanes 9–12 of the beta-catenin blot. BioMed Central has been advised by the authors’ institution that investigations by the Research Integrity Committee of the New York State Institute for Basic Research in Developmental Disabilities, the Research Foundation for Mental Hygiene Governance Committee and the Research Foundation for Mental Hygiene Board of Directors concluded that this article should be removed from the scientific literature. W Ted Brown, Ashfaq M Sheikh and Zujaja Tauqeer have agreed with this retraction. We have been unable to contact Fujiang Cao, Ailan Yin, Guang Wen, Mazhar Malik, Amenah Nagori, Michael Schirripa, Frank Schirripa, George Merz and Shiqing Feng.
